# Dynamic contrast-enhanced MRI detects acute radiotherapy-induced alterations in mandibular microvasculature: prospective assessment of imaging biomarkers of normal tissue injury

**DOI:** 10.1038/srep29864

**Published:** 2016-08-08

**Authors:** Joint Head and Neck Radiotherapy-MRI Development Cooperative, Vlad C. Sandulache, Brian P. Hobbs, Abdallah S.R. Mohamed, Steven J. Frank, Juhee Song, Yao Ding, Rachel Ger, Laurence E. Court, Jayashree Kalpathy-Cramer, John D. Hazle, Jihong Wang, Musaddiq J. Awan, David I. Rosenthal, Adam S. Garden, G. Brandon Gunn, Rivka R. Colen, Nabil Elshafeey, Mohamed Elbanan, Katherine A. Hutcheson, Jan S. Lewin, Mark S. Chambers, Theresa M. Hofstede, Randal S. Weber, Stephen Y. Lai, Clifton D. Fuller

**Affiliations:** 1 Department of Head and Neck Surgery, UT MD Anderson Cancer Center, Houston, TX, USA; 2 Department of Biostatistics, UT MD Anderson Cancer Center, Houston, TX, USA; 3 Department of Radiation Oncology, UT MD Anderson Cancer Center, Houston, TX, USA; 4 Department of Imaging Physics, UT MD Anderson Cancer Center, Houston, TX, USA; 5 Department of Radiation Physics, UT MD Anderson Cancer Center. Houston, TX, USA.; 6 Athinoula A. Martinos Center for Biomedical Imaging, Massachusetts General Hospital/Division of Health Sciences & Technology, Massachusetts Institute of Technology, Charlestown, MA, USA; 7 Department of Radiation Oncology, Case Western Reserve University, Cleveland, OH, USA; 8 Department of Diagnostic Radiology, UT MD Anderson Cancer Center, Houston, TX, USA; 9 Department of Molecular and Cellular Oncology, UT MD Anderson Cancer Center, Houston, TX, USA; 10 Department of Clinical Oncology and Nuclear Medicine, Faculty of Medicine, University of Alexandria, Alexandria, Egypt.

## Abstract

Normal tissue toxicity is an important consideration in the continued development of more effective external beam radiotherapy (EBRT) regimens for head and neck tumors. The ability to detect EBRT-induced changes in mandibular bone vascularity represents a crucial step in decreasing potential toxicity. To date, no imaging modality has been shown to detect changes in bone vascularity in real time during treatment. Based on our institutional experience with multi-parametric MRI, we hypothesized that DCE-MRI can provide in-treatment information regarding EBRT-induced changes in mandibular vascularity. Thirty-two patients undergoing EBRT treatment for head and neck cancer were prospectively imaged prior to, mid-course, and following treatment. DCE-MRI scans were co-registered to dosimetric maps to correlate EBRT dose and change in mandibular bone vascularity as measured by K_trans_ and V_e_. DCE-MRI was able to detect dose-dependent changes in both K_trans_ and V_e_ in a subset of patients. One patient who developed ORN during the study period demonstrated decreases in K_trans_ and V_e_ following treatment completion. We demonstrate, in a prospective imaging trial, that DCE-MRI can detect dose-dependent alterations in mandibular bone vascularity during chemoradiotherapy, providing biomarkers that are physiological correlates of acute of acute mandibular vascular injury and recovery temporal kinetics.

Development of adaptive, personalized external beam radiotherapy (EBRT) regimens requires an improved understanding of normal tissue toxicity. For head and neck tumors treated with EBRT, osteoradionecrosis (ORN) of the mandible is one of the most devastating complications^1^,^2^,^3^,^4^. In the late 1980s, R.E. Marx put forth the prevailing pathophysiological model regarding the development of ORN, focused on hypovascularity, hypocellularity and hypoxia^5^,^6^. Although anatomic imaging using CT or MRI can identify ORN once it is manifest, no imaging modality to date has been shown to be able to detect EBRT-associated vascularity changes prior to ORN development^7^. Development of imaging modalities which can detect EBRT associated normal tissue toxicity such as altered bone vascularity is essential to improving clinical outcomes^8^,^9^.

DCE-MRI is a clinically available imaging method, which is ideally positioned to fulfill the clinical goals listed above. It can detect early-stage idiopathic osteonecrosis of the femur not otherwise visible on conventional MRI^10^. DCE-MRI parameters can be used to monitor bone healing secondary to trauma/fracture as well as chronic changes in bone health associated with age-related osteoporotic changes^11^,^12^,^13^. Most importantly, this approach has superior accuracy compared to conventional MRI^14^. DCE-MRI can also identify changes associated with development of bony metastasis and tumor response to treatment^15^,^16^,^17^.

We hypothesized that DCE-MRI can detect changes in mandibular bone vascularity at EBRT doses encountered in clinical practice. While DCE-MRI has been shown to be useful in tumor imaging, to date, no prospective data exist regarding MRI assessment of mandibular radiation injury, nor any indication of the potential for evaluation of short- to intermediate-term recovery of radiotherapy-attributable vascular insult. Consequently, in an effort to determine the feasibility of DCE-MRI for detection of acute radiotherapy injury and recovery/persistence, we undertook a prospective investigation of temporal kinetics and dose-dependent shifts in mandibular perfusion parameters using alteration of the volume transfer coefficient (K_trans_) and the volume of the interstitium (V_e_), a surrogate for vascular flow and edema, respectively, from pre-therapy baseline as a candidate tissue-damage biomarker. Specifically, we undertook the following specific experimental aims:

1)determine the feasibility of serial, standardized DCE-MRI mandibular data acquisition to identify vascular/perfusion alteration during and immediately after radiotherapy;

2a)characterize the temporal kinetics and natural history of the K_trans_ and V_e_ parameters within the mandibular region of interest (ROI) during treatment;

2b)determine whether identifiable dose-response profiles indicative of persistent/resolving K_trans_ and V_e_ alterations could be observed;

3)prospectively interrogate dose-response relationships between regional mandibular radiation dose and ΔK_trans_ and ΔV_e_.

## Results

### Patients

A total of 32 patients were included in the prospective arm of the study. Patient and tumor characteristics are summarized in Table 1. All tumors were positive for p16, and 31/32 patients were male. The patient cohort is reflective of the HPV-associated shift in patient demographics, including younger age of presentation (58 years) and decrease in tobacco exposure.

### Treatment

Treatment characteristics are summarized in Table 2. All patients were treated non-surgically. Patients were relatively evenly split between IMRT and IMPT. All patients underwent concurrent chemotherapy treatment, primarily consisting of weekly cisplatin (27/32 patients); 3 patients underwent induction chemotherapy.

### Imaging of mandibular vascularity

**Figure 1:**
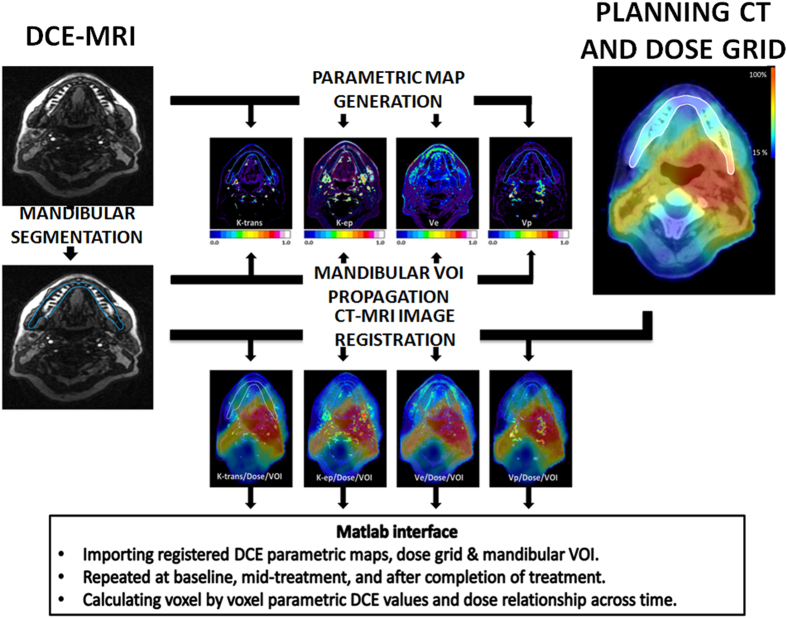
Imaging Workflow. Registration of DCE-MRI parametric maps to radiation dose grid across time for each patient.

**Figure 2:**
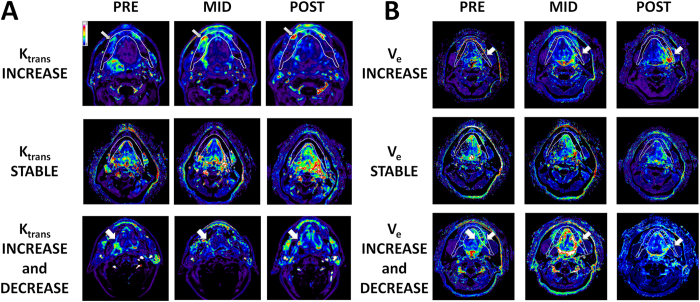
DCE-MRI Detects Focal Vascularity Changes During EBRT. Evaluation of DCE-MRI acquired K_trans_ across the entire mandibular volume allows for identification of geographically distinct perturbations which can then be correlated to the dosimetric map for each individual patient. **(A)** Arrows in top and bottom rows identify the area of altered K_trans_. Middle row demonstrates no appreciable change in K_trans_ across the entire mandibular volume regardless of administered EBRT dose. **(B)** Arrows in top and bottom rows identify the area of altered V_e_. Middle row demonstrates no appreciable change in V_e_ across the entire mandibular volume regardless of administered EBRT dose. Panels A and B represent distinct patients.

All patients underwent imaging according to our current institutional algorithm (Fig. 1). DCE-MRI derived data (K_trans_ and V_e_) was overlaid onto the anatomic dosimetric map in order to generate a voxel-by-voxel analysis. This resulted in a dataset of values corresponding to the entire administered dose gradient. As shown in Fig. 2, this allowed us to detect and quantify dose-dependent changes in K_trans_ and V_e_ for each individual patient in the cohort.

Summary of the inter-subject variance of the imaging parameters by scan time and dose are provided in Table 3.

**Figure 3:**
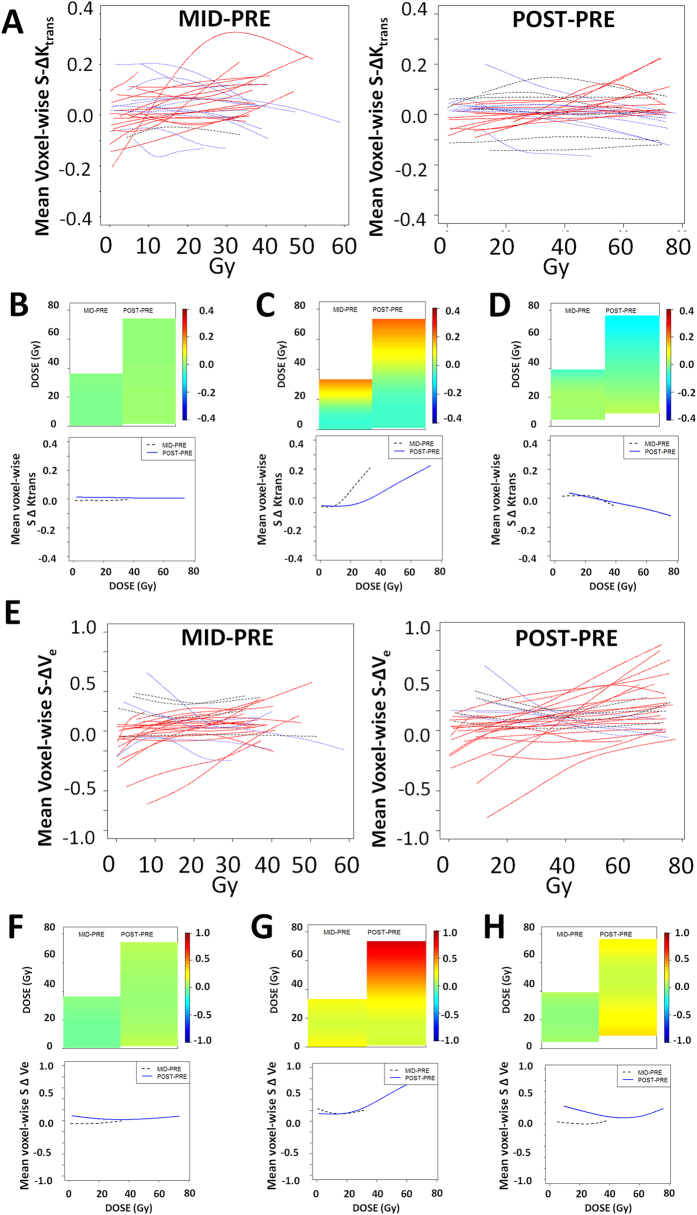
DCE-MRI Detects Dose-Dependent Vascularity Changes During EBRT. (**A**) Composite histogram of DCE-MRI acquired K_trans_ values across the entire mandibular region of interest (ROI). K_trans_ analysis was performed on a voxel-wise basis across the entire mandibular ROI and at specific EBRT doses for each patient. The figures depict the voxel-wise mean S ΔK_trans_ by dose trajectories estimated at two time points using cubic spline analysis. Red (blue) lines indicate patients which demonstrate a dose-dependent increase (decrease) in mean S ΔK_trans_ from pre-treatment values. Grey lines represent patients without a dose-dependent change in mean S ΔK_trans_. (**B**) Heatmap with color indicating the extent of vascular modulation and corresponding mean S ΔK_trans_ trajectories demonstrating a stable K_trans_ value set across the irradiation period regardless of dose. (**C**) Heatmap and S ΔK_trans_ trajectories demonstrating a dose-dependent increase in K_trans_ value set across the irradiation period. (**D**) Heatmap and S ΔK_trans_ trajectories demonstrating a dose-dependent decrease in K_trans_ value set across the irradiation period. (**E**) Composite histogram of DCE-MRI acquired Ve values across the entire mandibular region of interest (ROI). Ve analysis was performed on a voxel-wise basis across the entire mandibular ROI and at specific EBRT doses for each patient. The figures depict the voxel-wise mean S ΔVe_s_ by dose trajectories estimated at two time points using cubic spline analysis. Red (blue) lines indicate patients which demonstrate a dose-dependent increase (decrease) in mean S ΔVe from pre-treatment values. Grey lines represent patients without a dose-dependent change in mean S ΔVe. (**F**) Heatmap with color indicating the extent of vascular modulation and corresponding mean S ΔVe trajectories demonstrating a stable Ve value set across the irradiation period regardless of dose. (**G**) Heatmap and S ΔVe trajectories demonstrating a dose-dependent increase in Ve value set across the irradiation period. (**H**) Heatmap and S ΔVe trajectories demonstrating a dose-dependent decrease in Ve value set across the irradiation period. **MID-PRE** = change across EBRT dose between PRE-irradiation scan and MID-irradiation scan. **POST-PRE** = change across EBRT dose between PRE-irradiation scan and POST-irradiation scan.

The primary analysis was conducted by measuring absolute and relative changes in K_trans_ and V_e_ values using two points of comparison. As shown in Fig. 3A, the relative change in K_trans_ and V_e_ values between the PRE and MID (course) DCE-MRI scans was calculated as a function of EBRT dose. Similarly, the relative change in K_trans_ and V_e_ values between the PRE and POST (treatment) DCE-MRI scans was calculated as a function of EBRT dose.

### Mid-treatment vascularity changes

We identified a large subset of patients which demonstrated measurable changes in K_trans_ (97%) and V_e_ (87%) at the midpoint of treatment. Among these patients 58% demonstrated a dose dependent increase (estimated >0.5% increase in vascular parameter per unit increase in dose) in K_trans_ and 39% demonstrated a dose dependent decrease (estimated >0.5% decrease in vascular parameter per unit increase in dose) in K_trans_. Mean value for increasing K_trans_ was 12.5 min^−1^ (SD 14), while the mean value for decreasing K_trans_ was 3.8 min^−1^ (SD 4.1) (p-value for a difference among the 3 cohorts <0.0001). Sixty-eight percent of patients demonstrated a dose-dependent increase (see above) in V_e_ and 19% demonstrated a dose dependent decrease (see above) in V_e_. Sixty-five percent of patients demonstrated concordance between K_trans_ and V_e_ dose dependent trends. Figure 3 illustrates K_trans_ (3A) and V_e_ (3E) changes over time according to EBRT dose along with sample individual patient heatmaps for each parameter (K_trans_ Fig. 3B–D; V_e_ Fig. 3F–H). Mean value for increasing V_e_ was 5.0 (SD 7.8), while the mean value for decreasing V_e_ was 3.6 (SD 2.8) (p-value for a difference among the 3 cohorts <0.0001).

### Post-treatment vascularity changes

We identified a large subset of patients which demonstrated measurable changes in K_trans_ (80%) and V_e_ (87%) at the completion of treatment (Fig. 3). Among these patients 43% demonstrated a dose dependent increase in K_trans_ and 37% demonstrated a dose-dependent decrease in K_trans_. Mean value for increasing K_trans_ was 7.5 min^−1^ (SD 9.5), while the mean value for decreasing K_trans_ was 4.2 min^−1^ (SD 4.8) (p-value for a difference among the 3 cohorts <0.0001). Seventy percent of patients demonstrated a dose-dependent increase in V_e_ and 17% demonstrated a dose dependent decrease in V_e_. Fifty percent of patients demonstrated concordance between K_trans_ and V_e_ dose dependent trends. Mean value for increasing V_e_ was 15.5 (SD 31.9), while the mean value for decreasing V_e_ was 1.7 (SD 0.7) (p-value for a difference among the 3 cohorts <0.0001).

One patient developed ORN during the study period. This patient initially exhibited a dose-dependent increase in both K_trans_ and V_e_ at the midpoint of treatment, with subsequent decrease in both parameters at the post-treatment scan.

### IMRT vs IMPT analysis

The distribution of patients across the two irradiation modalities was relatively balanced (IMPT n = 19, IMRT n = 13). The distribution of patients across the two irradiation modalities was relatively balanced (IMPT n = 19, IMRT n = 13). Similar percentages of patients treated with IMPT demonstrated a dose dependent increase in V_e_ compared to patients treated with IMRT (MID-PRE 70% vs 50%; POST-PRE 72% vs 67%); neither difference was statistically significant (Z-test) (Table 4). Similar percentages of patients treated with IMPT demonstrated a dose dependent increase in K_trans_ at the mid-point of treatment compared to IMRT treated patients (68% vs 42%) but this relationship was reversed at the end of treatment (39% vs 50%); neither difference was statistically significant (Z-test).

## Discussion

Over the last two decades, we have observed an explosive increase in the frequency of oropharyngeal squamous cell carcinoma (OPSCCA) cases, driven primarily by the oncogenic effect of HPV^18^,^19^,^20^,^21^. In contrast to traditional OPSCCA patients, patients whose tumors are driven by HPV are generally younger and healthier. Due to excellent oncologic outcomes in this OPSCCA subset, patients are expected to live long, cancer-free lives following completion of treatment^18^. As a result, limiting normal tissue toxicity associated with EBRT is crucial to providing good quality of life following oncologic cure. Osteoradionecrosis (ORN) of the mandible is a devastating complication associated with EBRT treatment of head and neck cancers^22^,^23^,^24^,^25^. Increased utilization of IMRT was initially expected to eliminate the development of ORN, by sparing the mandible of high EBRT doses. However, over the last several years, multiple investigators, including our group, have found that ORN rates can exceed 5% of treated head and neck cancer patients even in cohorts treated exclusively with IMRT^22^,^25^.

Our current understanding of ORN development holds that hypoperfusion and hypovascularity decrease bone health and the ability to appropriately react to trauma and infection, and serve as either surrogates or harbingers of temporally subsequent fibrotic processes^6^. It is thought that radiation will affect the endosteal blood flow due at least in part to intimal proliferation of the inferior alveolar artery and soft tissue fibrosis that may diminish periosteal blood flow. The end result is decreased vascularity to the cortical and medullary bone. In the present work we sought to identify potential imaging biomarkers of altered bone perfusion and develop a mechanism for identification of patients at risk late sequelae such as ORN. We focused our attention on K_trans_ and V_e_, DCE-MRI parameters which are considered good surrogates for vascular flow and edema respectively. Based on our understanding of EBRT effects of tissue vascularity, we expected that both parameters would be altered during EBRT treatment in a dose dependent manner. Data summarized above demonstrate for the first time that, vascular flow, ascertained via K_trans_ is altered in a dose-dependent fashion during and immediately following EBRT treatment. In a similar fashion, edema as measured via V_e_ is altered by ERBT in a dose-dependent fashion. The finding that in the majority of patients both parameters exhibit a dose dependent increase at both time points suggests both an increase in vascular flow and potential radiation induced damage to mandibular vasculature resulting in increased edema.

DCE-MRI is already utilized in the diagnosis and monitoring of solid tumors^15^,^17^. It has demonstrated utility in the monitoring of tumor vascularity^26^ and has been used to characterize bone vascularity in the setting of acute trauma and bone repair^10^,^11^,^12^,^13^,^14^,^16^,^17^. Because of its sensitivity to perturbations in tissue vascularity, K_trans_ and V_e_ values vary significantly in the context of various pathologic conditions secondary to onset of inflammation, damage to endothelial cells and vessel wall integrity, and vessels occlusion and fibrosis. The data summarized above are consistent with this multifaceted impact.

In this study, quality assurance was performed and reviewed routinely by qualified medical physicists to ensure high quality MR images can be consistently obtained during this longitudinal study. Using the workflows described, we were able to show the feasibility of longitudinal acquisition of serial pre-, mid-, and post-EBRT DCE-MRIs for all patients in a strict positioning and immobilization as during radiation treatment which ensured superior quality images with no motion artifacts and easiness of registration to planning CT and dose grid to monitor dose-dependent vascular changes. In general, the interpretation of vascular flow as K_trans_ is inappropriate because K_trans_ calculated by extended Tofts model generally represents a mixture of vascular flow and vascular leakiness. A two-compartment exchange model could be a more appropriate way to estimate perfusion and capillary permeability^27^. However, our study investigates a weakly vascularized tissue namely, mandible bone. In this case, we believe that K_trans_ is appropriately considered to mainly reflect vascular flow^28^. Moreover, the extended Tofts model improves precision in the estimates of V_e_ which in this context is interpreted as a measure of overall tissue edema.

Additionally, we demonstrated that a majority of patients undergoing EBRT for OPSCCA in our series demonstrate detectable mid-treatment perturbations in mandibular K_trans_ and V_e_, in a dose-dependent manner which generally persists at the completion of treatment. The ability to capture this information during the in-treatment time frame represents a novel implementation of clinical DCE-MRI imaging in addition to tumor assessment. Whether these acute vascularity alterations predict eventual ORN development remains unclear. To date only one patient from this cohort has developed clinical ORN. Of note, in this patient, both parameters demonstrated a dose dependent decrease at the time of post treatment imaging. A minority of patients demonstrated decreasing K_trans_ and V_e_ as a function of EBRT dose. It is possible that this subset of patients is at increased risk of ORN development. One limitation of the current study is the lack of substantial data regarding EBRT associated normal tissue clinical sequelae (i.e. ORN development). This prevents correlation of the EBRT induced vascularity alterations measured acutely with long term clinical parameters with the exception of the above described patient. Institutionally, we have demonstrated a 6–7% rate of frank ORN, and as the majority of ORN cases develop in the first three years following irradiation, the current cohort is still relatively limited with respect to this clinical entity^25^. Nonetheless, this dataset represents, to our knowledge, the first prospective assessment of DCE-MRI biomarkers for dose-dependent acute mandibular vascular changes. As such, this work serves as template for further longitudinal investigation of MRI in normal tissue toxicity assessment in head and neck cancer patients.

The primary treatment paradigm for HPV-associated OPSCCA consists of EBRT, in the form of IMRT or more recently, IMPT^21^. Given the increasing utilization of IMPT in the treatment of head and neck cancers at large academic centers, the current cohort was deliberately balanced in its inclusion of irradiation methodology to provide data that can be utilized over the coming years, while results from the current prospective imaging trial investigated acute mandible injury, we will continue to capture intermediate and late effects (such as patient-reported outcomes and rate of ORN development) as the current Phase II/III trial continues accrual. It is our hypothesis that acute, dose-dependent K_trans_ and V_e_ perturbations (ΔK_trans_ and ΔV_e_) can act as harbingers of long-term alterations in mandibular bone perfusion. Whether this translates into an increased risk of ORN development is the subject of a prospective institutional clinical protocol designed to ascertain the natural history of ORN and identify factors which predispose patients to ORN development.

## Conclusions

**This study is the first to demonstrate that DCE-MRI can detect in-treatment changes in bone perfusion that correlate with EBRT dose-escalation in a subset of head and neck cancer patients.** We demonstrate that DCE-MRI can feasibly identify and monitor *in vivo* EBRT-induced acute changes in mandibular bone through alterations in K_trans_ and V_e_ for patients undergoing treatment for OPSCCA. K_trans_ and V_e_ may be considered imaging biomarkers for subclinical radiation injury and recovery processes in the acute mid- and post-therapy setting. Future efforts aimed at validation of these parameters as measured by DCE-MRI for prediction and detection of late radiotherapy mandibular sequelae are warranted.

## Materials and Methods

### Patients

The study was approved by the University of Texas MD Anderson Cancer Center institutional review board (NCT01893307). All study methods were carried out in accordance with the IRB-approved guidelines. Written informed consent was obtained from all patients enrolled in this study. Patients were prospectively enrolled between October 2013 and May 2015. Inclusion criteria were: age >18 years, stage III-IV human papilloma virus (HPV) positive oropharynx SCC (American Joint Committee on Cancer staging), eligibility for definitive chemoradiotherapy, Eastern Cooperative Oncology Group performance status of 0–2. Exclusion criteria were: definitive resection of primary tumor, prior cancer diagnosis, prior radiotherapy to the head and neck, contraindications to gadolinium-based agents. Demographics, tobacco and alcohol exposure, and patient clinical-pathologic history were reviewed. Patients were considered to have a smoking history if they were current smokers, or former smokers with greater than 10 pack/year history.

### Imaging

Patients underwent: 1) baseline scan within 1 week prior to treatment, 2) mid-treatment scan 3–4 weeks after treatment initiation and 3) scan 6–8 weeks after last treatment EBRT fraction. MRI was performed using a custom immobilization method^29^ and a 3.0T Discovery 750 MRI scanner (GE Healthcare, Waukesha, WI) with laterally placed 6-element flex coils centered on the base of the tongue with immobilization devices (Klarity Medical Products, Newark, OH). Mid- and post- treatment data were indexed to pre-treatment data acquired with the patient on a flat insert table (GE Healthcare) using the same immobilization devices as those used for daily image-guided therapy (individualized head and shoulder mask, customized head support, intraoral tongue-immobilizing/swallow-suppressing dental stent).

Geometrical scan parameters (field of view (FOV) = 25.6 cm, slice number = 30, slab thickness = 12 cm, pixel size = 1 mm × 1 mm in-plane) were prescribed for a standardized spatial region encompassing the palatine process region cranially to the cricoid cartilage caudally for all scans. T2w and T1w axial images were acquired using a fast spin-echo sequence (T2w: repetition time/echo time (TR/TE) = 3.6 s/100 ms, echo train length (ETL) = 16; pre-contrast T1w: TR/TE = 630/7 ms, ETL = 2; post-contrast T1w with fat saturation: TR/TE = 592/7 ms, ETL = 2). Prior to DCE MRI, T1 mapping was performed using a total of 6 variable flip angle (FA) 3D spoiled gradient recalled echo (SPGR) sequence (FA = 2°, 5°, 10°, 15°, 20°, and 25°; TR/TE = 5.5/2.1 ms, NEX = 0.7, spatial resolution = 2 mm × 2 mm × 4 mm). The DCE-MRI acquisition consisted of a 3D SPGR sequence to gain sufficient signal-to-noise ratio (SNR), contrast, and temporal resolution. The following scan parameters were used: FA = 15°, TR/TE = 3.6/1 ms, NEX = 0.7, spatial resolution 2 mm × 2 mm × 4 mm, temporal resolution = 5.5s, number of temporal position = 56, pixel bandwidth = 326 Hz, parallel imaging (ASSET) with acceleration factor 2. Images were acquired every 5.5 s for a total of 5 minutes. Gadopentetate dimeglumine (Magnevist, Bayer Healthcare Pharmaceuticals) was administered (dose 0.1 mmol/kg at a rate of 3 mL/s) followed by a 20 ml saline flush, via a power injector (Spectris MR Injector, MedRad, Pittsburgh Pa) at a rate of 3 mL/s. Total acquisition time was ~8 min.

We used the extended Tofts model pharmacokinetics analysis. T1 maps were calculated using the Nonlinear Least Squares Regression curve fitting in ImageJ^30^ (NIH, Bethesda, MD, USA). Supplementary Figure 1 shows an example of a pre-, mid-, and post-treatment T1 map. The T1-maps and DCE-MRI data, along with a boot-strapped population arterial input function (AIF) measured from a region of interest in the carotid artery (Supplementary Figure 2) were imported to NordicICE software (Version 2.3.14; NordicNeuroLab, Bergen, Norway). Data were used in the computation of the DCE-MRI parametric maps, with motion correction to enhance the quality of DCE map computation, with co-registration of post-contrast T1 and DCE maps using manually segmented ROIs of the mandible and maxilla for obtaining pharmacokinetics parameters, which include maps for the parametric volume transfer constant (K_trans_, [min^−1^] representing transfer of the contrast agent from the vascular space to the extravascular, extracellular space (EES)) and interstitial volume (V_e_, [%])^31^.

Planning CT and dose grids were retrieved and registered to DCE-MRI using commercial software solution (Velocity AI, 3.0.1, Atlanta, GA). Voxel-by-voxel dose-K_trans_ mapping for mandibular ROIs in each time point was then obtained using in-house Matlab code (Matlab, Mathworks, 2013a). The imaging workflow is illustrated in Fig. 1^32^.

### Statistical analysis

A two-stage data analysis plan was implemented separately for each DCE-MRI parameter (K_trans_ and V_e_) and each patient to first characterize the extent of change in mandibular vascularity from the PRE-irradiation scan to MID-irradiation and POST-irradiation scans using (the DCE-MRI parametric maps), then estimate the extent to which an alteration was attributable to EBRT using the co-registered dosimetric maps. To ensure that the observed differences in the numerical values of the DCE-MRI parameters were comparable between PRE to MID-irradiation and PRE to POST-irradiation scans, statistical analyses considered the standardized difference (SΔ) that is obtained by dividing the observed absolute inter-scan difference for each parameter by its corresponding sample coefficient of variation when estimated using all voxels contributed by the PRE-irradiation scan. After computing the standardized differences in the DCE-MRI parameters, SΔK_trans_ and SΔV_e_, between PRE and follow-up scans in the first stage, functional data analytic techniques were used to obtain smooth estimates of the SΔK_trans_ and SΔV_e_ trajectories over the continuous dose domain. Assuming that the observed voxel-wise standardized differences represent exchangeable Gaussian-distributed random variables, penalized cubic piecewise polynomial regression was used to characterize the induced functional relationships between EBRT dose and SΔK_trans_ and SΔV_e_ for each patient at each imaging follow-up time point^33^,^34^. Patients exhibiting EBRT-induced alterations in mandibular bone vascularity were identified for each DCE-MRI parameter from the resultant derivatives of the estimated mean trajectories. Patients experiencing a greater than 0.5% mean standardized increase per unit increase in dose were determined to have exhibited increasing trends. Patients with an estimated greater than 0.5% mean standardized reduction per unit increase in dose were considered to have experienced decreasing trends. An EBRT induced change in mandibular vascularity was determined to be absent for patients that exhibited neither increasing nor decreasing trends. The resultant mean derivatives are reported for each DCE-MRI parameter at each follow-up scan time. Additionally, to summarize the extent to which the derivatives varied among the three trajectory cohorts we report the corresponding p-values obtained from the Kruskal-Wallis test. The statistical software R (R Development Core Team, http://www.r-project.org) version 3.2.1 was used for statistical analysis.

## Additional Information

**How to cite this article**: Joint Head and Neck Radiotherapy-MRI Development Cooperative. Dynamic contrast-enhanced MRI detects acute radiotherapy-induced alterations in mandibular microvasculature: prospective assessment of imaging biomarkers of normal tissue injury. *Sci. Rep.* **6**, 29864; doi: 10.1038/srep29864 (2016).

## Supplementary Material

Supplementary Information

## Figures and Tables

**Table 1:** Patient characteristics.

Patients		32
Average age (yr)		59.03
Gender	male	31
	female	1
Race	white	29
	black	1
	other	2
<10pkyr	20	
	>10pkyr	9
Site	oropharynx	32
Subsite	tonsil	16
	base of tongue	15
	other	1
T stage	1	2
	2	12
	3	9
	4	9
N stage	0	2
	1	4
	2a	3
	2b	12
	2c	12
	3	0
M stage	0	32
	1	0

**Table 2:** Treatment characteristics.

Radiation	IMRT	13
	IMPT	19
Induction chemotherapy	yes	3
	no	29
Concurrent chemotherapy	yes	32
	no	0

**Table 3:** Summary of inter-subject variance in K_trans_ and V_e_ parameters by scan time and radiation dose.

PARAMETER	SCAN Time	DOSE	SD	MEDIAN	IQR
K_trans_ (min^−1^)	PRE		2.28	0.08	0–0.37
	MID	10 Gy^1^	2.57	0.09	0–0.44
		30 Gy^2^	1.77	0.12	0–0.50
	POST	10 Gy^1^	1.28	0.10	0–0.41
		30 Gy^2^	1.84	0.10	0–0.40
V_e_ (%)	PRE		1.30	0.00	0–0.96
	MID	10 Gy^3^	1.37	0.00	0–1.07
		30 Gy^4^	1.41	0.15	0–1.12
	POST	10 Gy^3^	1.40	0.19	0–1.31
		30 Gy^4^	1.46	0.06	0–1.23

IQR = Inter Quartile Range.

SD = Standard deviation.

^1^K_trans_ for doses 9~11Gy.

^2^K_trans_ for doses 29~31Gy.

^3^V_e_ for doses 9~11Gy.

^4^V_e_ for doses 29~31Gy.

**Table 4:** K_trans_ and V_e_ value perturbations across EBRT modalities.

MID-PRE Ktrans			
	ALL	IMRT	IMRT
INCREASING	18	5	13
DECREASING	12	6	6
FLAT	1	1	0
**POST-PRE Ktrans**			
INCREASING	13	6	7
DECREASING	11	3	8
FLAT	6	3	3
**MID-PRE V**_**e**_			
INCREASING	21	6	15
DECREASING	6	4	2
FLAT	4	2	2
**POST-PRE Ve**			
INCREASING	21	8	13
DECREASING	5	3	2
FLAT	4	1	3
