# Supplementary material for: Dynamic contrast-enhanced MRI detects acute radiotherapy-induced alterations in mandibular microvasculature: prospective assessment of imaging biomarkers of normal tissue injury
Source: Sci Rep. 2016 Aug 8;6:29864. doi: 10.1038/srep29864 (PMC4976364; doi:10.1038/srep29864)
Supplement: Supplementary Information [file srep29864-s1.pdf]

Supplementary Figures

Dynamic contrast-enhanced MRI detects acute radiotherapy-induced alterations in mandibular microvasculature: prospective assessment of imaging biomarkers of normal tissue injury

Joint Head and Neck Radiotherapy-MRI Development Cooperative.

Contributing authors:
Vlad C. Sandulache [1], Brian P. Hobbs [2], Abdallah S.R. Mohamed [3], Steven J. Frank [3], Juhee Song [2], Yao Ding [4], Rachel Ger [X], Laurence E. Court [X], Jayashree Kalpathy-Cramer [5], John D. Hazle [4], Jihong Wang [4], Musaddiq J. Awan [6], David I. Rosenthal [3], Adam S. Garden [3], G. Brandon Gunn [3], Rivka R. Colen [7], Nabil El-shafeey [7], Mohamed Elbanan [7], Katherine A. Hutcheson [1], Jan Lewin [1], Mark S. Chambers [1], Theresa M. Hofstede [1], Randal S. Weber [1], Stephen Y. Lai [1,8]*, Clifton D. Fuller [3]*

1- Department of Head and Neck Surgery, UT MD Anderson Cancer Center, Houston, TX; 2- Department of Biostatistics, UT MD Anderson Cancer Center, Houston, TX; 3- Department of Radiation Oncology, UT MD Anderson Cancer Center, Houston, TX; 4- Department of Imaging Physics, UT MD Anderson Cancer Center, Houston, TX; X- Department of Radiation Physics, UT MD Anderson Cancer Center; 5- Athinoula A. Martinos Center for Biomedical Imaging, Massachusetts General Hospital/ Division of Health Sciences & Technology, Massachusetts Institute of Technology, Charlestown, MA; 6- Department of Radiation Oncology, Case Western Reserve University, Cleveland, OH; 7- Department of Diagnostic Radiology, UT MD Anderson Cancer Center, Houston, TX; 8- Department of Molecular and Cellular Oncology, UT MD Anderson Cancer Center, Houston, TX

*Co-corresponding authors.

Corresponding authors:
Stephen Y. Lai MD, PhD
The University of Texas MD Anderson Cancer Center
1515 Holcombe Blvd.
Unit Number: 1445
Houston, TX 77030
Phone: (713) 792-6528
Fax: (713) 794-4662
E-mail: sylai@mdanderson.org

Clifton D. Fuller MD, PhD
The University of Texas MD Anderson Cancer Center
1515 Holcombe Blvd.
Unit Number: 0097
Houston, TX 77030
Phone: (713) 792-3471
Fax: (713) 563-2366
E-mail: CDFuller@mdanderson.org

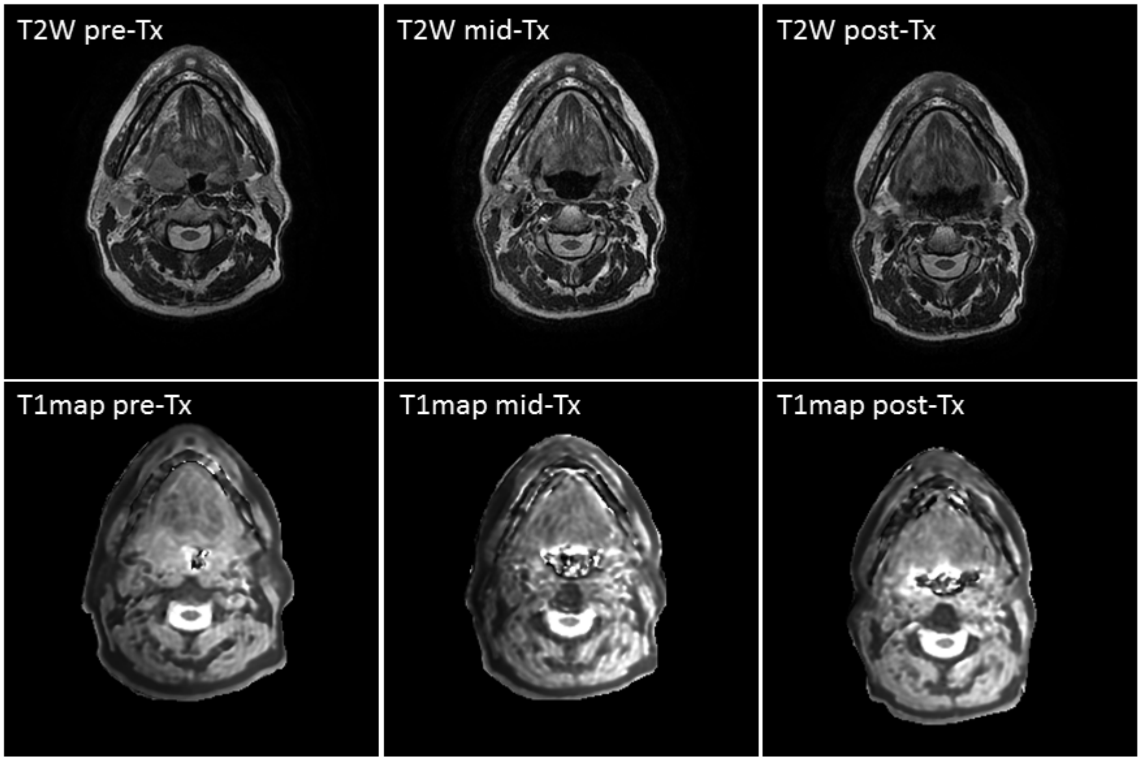

Supplementary Figure 1. Example of pre-, mid, and post-treatment T1 map for a patient with T4, N2b, M0 tonsil squamous cell carcinoma. The upper panel shows the T2 images and the lower panel shows the T1 maps.

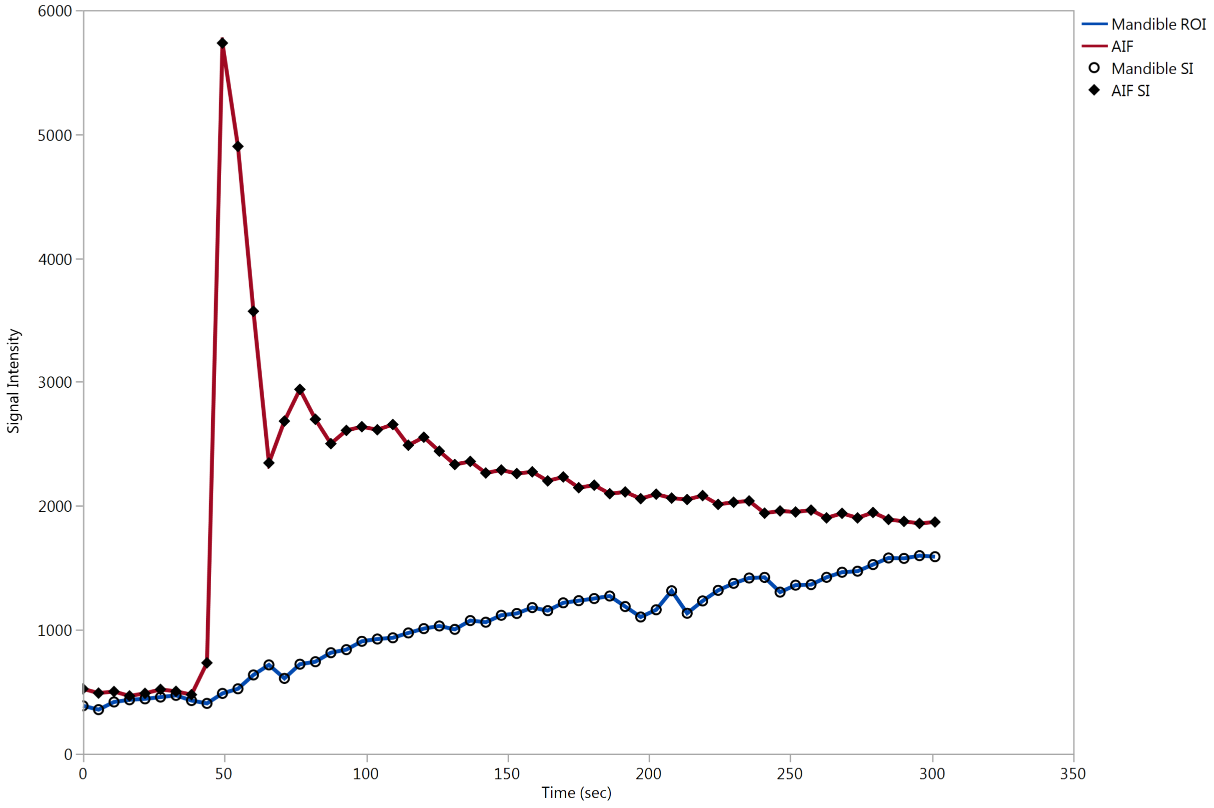

Supplementary Figure 2. Example of a mandibular region of interest signal intensity changes against time compared to the utilized standard arterial input function (AIF).
